# Structural Behavior of RC Column Confined by FRP Sheet under Uniaxial and Biaxial Load

**DOI:** 10.3390/polym14010075

**Published:** 2021-12-25

**Authors:** Huynh-Xuan Tin, Ngo-Thanh Thuy, Soo-Yeon Seo

**Affiliations:** 1 Faculty of Civil Engineering, University of Transport and Communications, No. 3 Cau Giay Street, Lang Thuong Ward, Dong Da District, Hanoi 11512, Vietnam; tinhx_ph@utc.edu.vn (H.-X.T.); thuynt_ph@utc.edu.vn (N.-T.T.); 2 Department of Architectural Engineering, Korea National University of Transportation, Chungju 27469, Korea

**Keywords:** structural behavior, uniaxial and biaxial load, fully and partially CFRP-confined reinforced concrete columns

## Abstract

Various researches have been performed to find an effective confining method using FRP sheet in order to improve the structural capacity of reinforced concrete column. However, most of these researches were undertaken for the columns subjected to concentric compressive load or fully confined RC columns. To date, it remains hard to find studies on partially FRP-confined RC columns under eccentric load. In this manner, an experimental investigation was carried out to assess the performance of rectangular RC column with different patterns of CFRP-wrap subject to eccentric loads in this paper. The experiment consists of fourteen mid-scale rectangular RC columns of 200 mm × 200 mm × 800 mm, including five controlled columns and nine CFRP-strengthened ones. All CFRP-strengthened columns were reinforced with one layer of vertical CFRP sheet with the main fiber along the axial axis at four sides, then divided into three groups according to confinement purpose, namely unconfined, partially CFRP-confined, and fully CFRP-confined group. Two loading conditions, namely uniaxially and biaxially eccentric loads, are considered as one of the test parameters. From the test of uniaxial eccentric load, partial and full CFRP-wraps provided 19% and 33% increased load-carrying capacity at an eccentricity-to-column thickness ratio (*e*/*h*) of 0.125, respectively, compared to controlled columns, and 8% and 11% at *e*/*h* = 0.25, respectively. For the partially CFRP-confined columns subjected to biaxial eccentric load with *e*/*h* = 0.125 and 0.25, the load-carrying capacities were improved by 19% and 31%, respectively. This means that the partial confinement with CFRP effectively improves the load-carrying capacity at larger biaxial eccentric load. It was found that the load-carrying capacity could be properly predicted by using code equations of ACI 440.2R-17 and Fib Bulletin 14 Guideline for the full CFRP-confined or partially CFRP-confined columns under uniaxial load. For partially CFRP-confined columns under biaxial loading, however, the safety factors using the Fib calculation process were 20% to 31% lower than that of uniaxially loaded columns.

## 1. Introduction

Reinforced concrete (RC) columns are critical structural components in building. In order to increase the load-bearing capacity and ductility of the columns, the concrete core is confined by providing lateral pressure. For improving the structural capacity of an existing column, steel jackets wrapped around the concrete cover have been used to confine RC columns. However, there are two main limitations for this method, namely the corrosion of steel and the need for prefabrication of specific column dimensions. Jacketing of RC columns with FRP (fiber reinforced polymer) sheet is an extension of the steel jacketing approach. The advantages of FRP include light weight, high stiffness, high resistance to corrosion, and easy adaptability to any shape of structural member so that it is flexibly applied through wet layup in the field.

For fully FRP-wrapped RC columns under eccentric compression, the load-carrying capacity and ductility of the columns have been improved significantly through several studies [1,2,3,4]. Chaallal and Shahawy [1] investigated the performance of reinforced concrete members strengthened with externally applied bidirectional CFRP material. From the experiment with the eccentric distance as the main variable, they found that the strength capacity of members improved significantly as a result of the combined action of the longitudinal and the transverse weaves of the bidirectional composite fabric. The maximum capacity gain achieved was slightly below 30% in pure compression, and over 54% in pure flexure. Eshghi and Zanjanizadeh [2] studied the seismic repair of damaged square reinforced concrete columns with poor lap splices, 90-degree hooks, and widely spaced transverse bars in plastic hinge regions using glass fiber reinforced plastic (GFRP) sheets. Three specimens of 150 × 150 × 1000 mm were tested in “as built” condition and retested after being repaired by GFRP sheets in critically stressed areas near the column footings. The test includes numerous reversed lateral cyclic loads with a constant axial load ratio. The results indicated that, by increasing the existing confinement in the column critical regions, ductility of repaired columns was improved from 12% to 113%. Rahai and Akbarpour [3] presented the results of an experimental study on rectangular RC columns strengthened with CFRP sheets under axial load and biaxial bending moment. Eight large-scale RC columns of 150 mm × 450 mm × 2400 mm were tested under bi-eccentric compressive loading up to failure. Investigation parameters include CFRP thickness of one, two, three, and four layers, fiber orientations of ±45, 0, 90, and their combination, and eccentricities in the direction of both weak axis and main axis. From the experiment, they found that a great improvement in moment strengthening up to 250%, and in compression strength up to 64% thanks to CFRP confinement. Hadi and Widiarsa [4], also studied the influence of the number of CFRP layers (one, two, and three layers), the magnitude of eccentricity (0, 20, and 25 mm) and the presence of vertical CFRP straps by testing sixteen specimens under eccentric loading. Results of this study showed that CFRP wrapping enhanced the load-carrying capacity (up to 18%) and ductility (up to 300%) of the columns under eccentric loading. Furthermore, the application of the vertical CFRP straps significantly improved the performance of the columns with large eccentricity.

FRP-confined concrete also enhanced the ductility, energy dissipation capacity, and strength of fully FRP-wrapped columns under seismic loads [5,6,7]. Manie et al. [8] found that retrofitting RC square columns by longitudinal fiber arrangement is only effective for columns with tension-controlled behavior, while transverse and combined longitudinal-transverse arrangements are more effective in enhancing the load bearing capacity of both the compression- and tension-controlled columns. Relative enhancements in axial resistance provided by fully FRP-wrapped circular columns were more significant under eccentric loading than in pure compression [9]. Recently, NadimiShahraki and Reisi [10] suggested an algorithm for the determination of the axial and bending capacity (uniaxial and biaxial) of RC strengthened columns by using various stress–strain curves of confined concrete presented by several researchers and the ACI code. From the comparison between predictions using the algorithm and experimental results, it was found that the axial and biaxial bending capacity of column strengthened with FRP wrapping could be well predicted.

According to the research results of previous researchers for partially FRP-confined RC columns, the strength and ductility of partially FRP-confined RC specimens can be significantly increased [11,12,13]. Barros and Ferreira [11] studied the effectiveness of the discrete confinement through concrete cylinders confined by distinct arrangements of strips of CFRP sheet under concentric load up to the failure point. From the evaluation on the influence of the width of the strip, distance between strips, number of CFRP layers per strip, CFRP stiffness, and concrete strength class, it was found that load carrying capacity and ductility of concrete specimens increase up to 3.27 and 5.02 times, respectively. Abdel-Hay [12] investigated the overall behavior of ten RC square columns. The main parameters studied in this research were the compressive strength of the upper part, the height of the upper poor concrete part, and the height of CFRP wrapped part of column under eccentric load. It was found that partially CFRP strengthening of square column gave good results in term of loading capacity, up to 1.3 times in compare with controlled column. The study of Lewangamage et al. [13] used 17 RC specimens of 150 mm × 150 mm × 350 mm with fully and partially CFRP confinement under concentric load. Although the volumetric ratio of CFRP was kept same for all partially confined columns, it was observed that, depending on the jacket location, the strength and ductility increments would vary. The experimental results showed considerable improvements in strength (up to 83% and 100% for fully and partially confined specimens, respectively) and ductility (up to 10 and 12.7 times for fully and partially confined specimens, respectively) in comparison with controlled specimens. Turğay et al. [14] focused on the investigation of the total effect of longitudinal and transverse reinforcement of large-scale square RC columns and figured out that partial CFRP-wrapping resulted an increase in ductility of longitudinal bars. Pham et al. [15] founded that a partial wrapping arrangement changes the failure modes of specimens and the angle of failure surface.

Unfortunately, most of these researches about partial FRP-wrapping were carried out with specimens subjected to concentric compressive load. To date, it remains hard to find studies on partially FRP-confined RC columns under eccentric load. Meanwhile, the prediction of the axial load-carrying capacity of fully FRP-confined RC columns has been evaluated in contemporary design guides for RC structures strengthened with FRP sheets such as CNR-DT 200 R1/2013 [16], ACI 440.2R-17 [17], and Fib Bulletin 14 [18]. However, the axial load-carrying capacity of partially confined RC columns with FRP sheet has been present only in Fib Bulletin 14 by suggesting a reduction factor to take into account the effect of partially wrapping with FRP sheet. Therefore, there has been a lack of theorical and experimental works concerning partially FRP-wrapped RC columns under concentric load. This study carried out an experimental investigation on the performance of a rectangular RC column with different patterns of CFRP-wrap subject to uniaxial and biaxial load. The primary objectives are to (1) evaluate the CFRP strengthening efficiency of partially and fully confined RC columns under uniaxial and biaxial load; and (2) study the existing guidelines on CFRP confinement design to establish the safety factor of the predicted load-carrying capacity against the experimental results.

## 2. Experimental Program

### 2.1. Specimen Design

**Figure 1:**
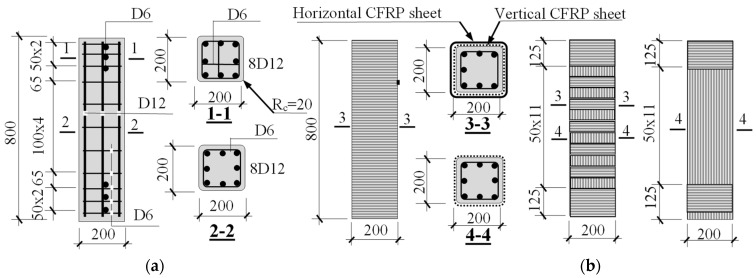
Detail of column specimens (in mm) (**a**) RC column; (**b**) reinforcement with FRP sheet.

Concentric and eccentric compressive test were planned. Fourteen mid-scale square RC columns with dimensions of 200 mm × 200 mm × 800 mm were fabricated as shown in Figure 1. These columns had identical reinforcements, including eight longitudinal 12-mm bars uniformly distributed around the perimeter (steel ratio of 2.28%). Stirrups with a diameter of 6 mm were used as a minimal requirement to minimize the additional confinement effect from the stirrups. As a result, the stirrup spacing of 100 mm was used and associated with the confining pressure (f_l_) of 0.06 MPa, less than 80% of concrete compressive strength (f_c_’), regarding ACI 440.2R-17 [17]. Stirrups with a smaller spacing of 50 mm and 3 steel meshes (6 mm diameter with a spacing of 50 mm) were placed near two ends of the columns, and 125 mm-width CFRP sheets were also wrapped at the two ends of the columns under eccentric load to avoid local damage.

**Figure 2:**
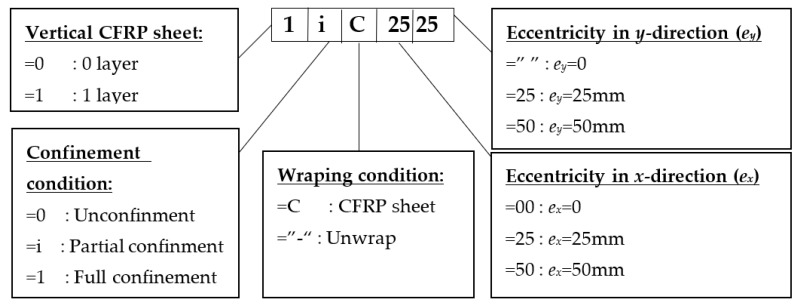
Description of the columns’ name.

The experiment includes 5 controlled columns and 9 CFRP-strengthening ones. All columns were cured at a temperature between 26 and 30 °C and humidity between 60% and 80%, within the manufacturer’s recommendations. After 28 days of curing, all controlled columns were tested and used as the reference columns, and 1 layer of vertical CFRP sheet with the main fiber along the axial axis at four sides was applied to all CFRP-strengthening columns. Then, these 9 strengthened columns were divided into three groups according to confinement purpose, namely unconfined (3 columns), partially CFRP-confined (4 columns), and fully CFRP-confined (2 columns). After 7 days of adhesive curing, 3 unconfined columns were tested. Meanwhile, partially and fully CFRP-confined columns were partially and fully wrapped for concrete confinement investigation, respectively. The description of the columns’ names is represented in Figure 2.

### 2.2. Material Properities

**Figure 3:**
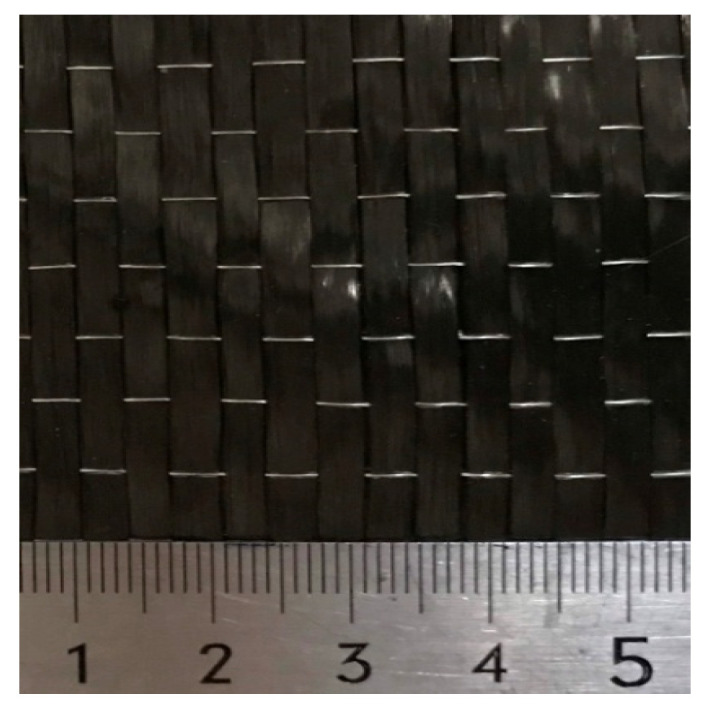
Unidirectional carbon-fiber sheet.

Concrete columns of M40 grade with the target design cubic-strength of 40 MPa were selected. The mixture design and its properties are tabulated in Table 1. The nominal diameters of longitudinal and transverse reinforcements were 12 mm and 6 mm, respectively. Carbotex UD 300 (Unidirectional Carbon Fibre Textile) by Adcos NV, Malle, Belgium as shown in Figure 3 and Carbotex Impreg (2-component solvent free epoxy system) by Adcos NV, Malle, Belgium were used in this study. The mechanical properties of reinforcements, CFRP sheets, and impregnation are tabulated in Table 2.

*F_adhesive,u_* and *E_adhesive_* are bond strenght and elastic module of adhesive, respectively, *f_fu_* and *ε_fu_* are ultimate tensile strength and strain, respectively, *t_f_* and *E_f_* are thickness and elastic module of CFRP, respectively, *f_u_*, *f_y_* and *E_s_* are ultimate, yield tensile strength and Elastic module of longitudinal rebars, respectively, *f_uw_*, *f_yw_* and *E_sw_* are ultimate, yield tensile strength and Elastic module of stirrup, respectively.

### 2.3. Strengthening with FRP Sheet

**Figure 4:**
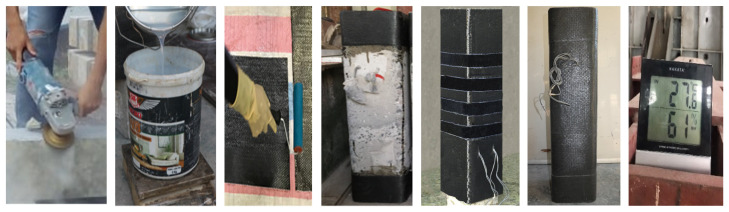
Procedure of strengthening with CFRP and curing.

Four corners of the columns were rounded off with a radius (R_c_) of 20 mm to eliminate the stress concentration and maximize the strengthening efficiency. The surface preparation was carefully performed according to the instructions of the supplier. CFRP sheets were wrapped around the perimeter of the columns with an overlapping-zone of 150 mm. Figure 4 represents the process of strengthening and curing.

### 2.4. Test Set Up and Installment of Equipment

**Figure 5:**
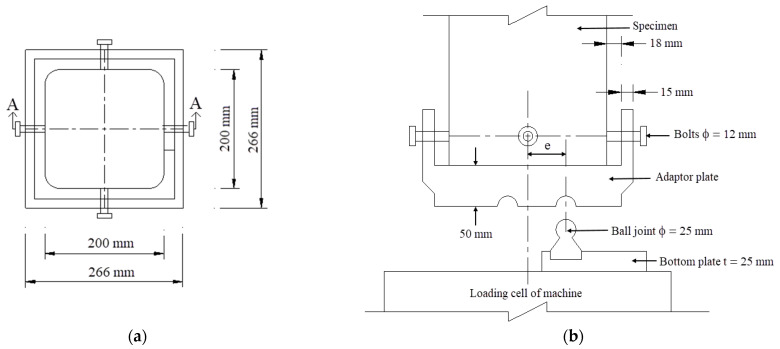
Eccentric loading mechanism; (**a**) Plan view; (**b**) Section A-A.

**Figure 6:**
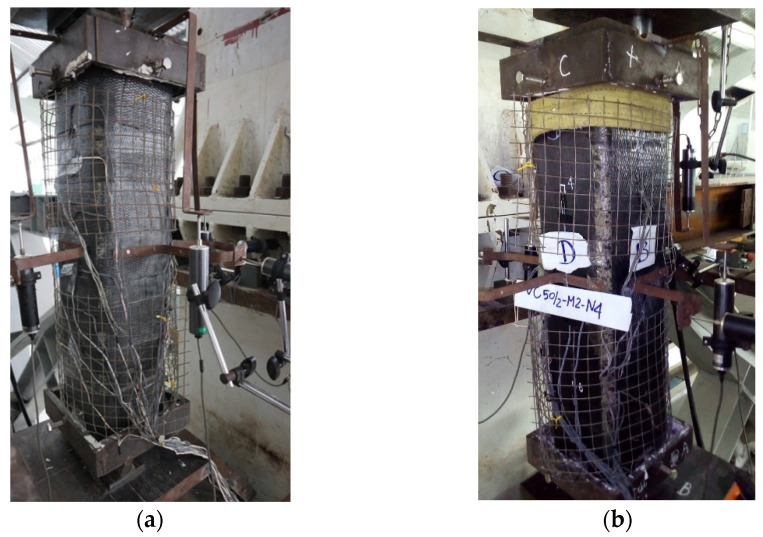

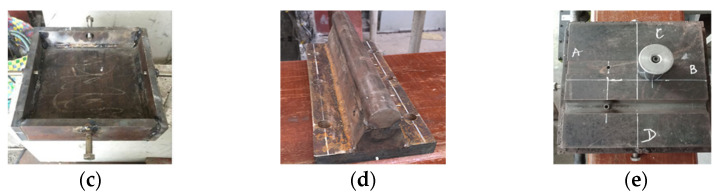
Setup of typical compression test: (**a**) Uniaxial loading; (**b**) Biaxial loading; (**c**) Adapter plate for uniaxial loading; (**d**) Ball joint for uniaxial loading; (**e**) Adapter plate and ball joint for biaxial loading.

**Figure 7:**
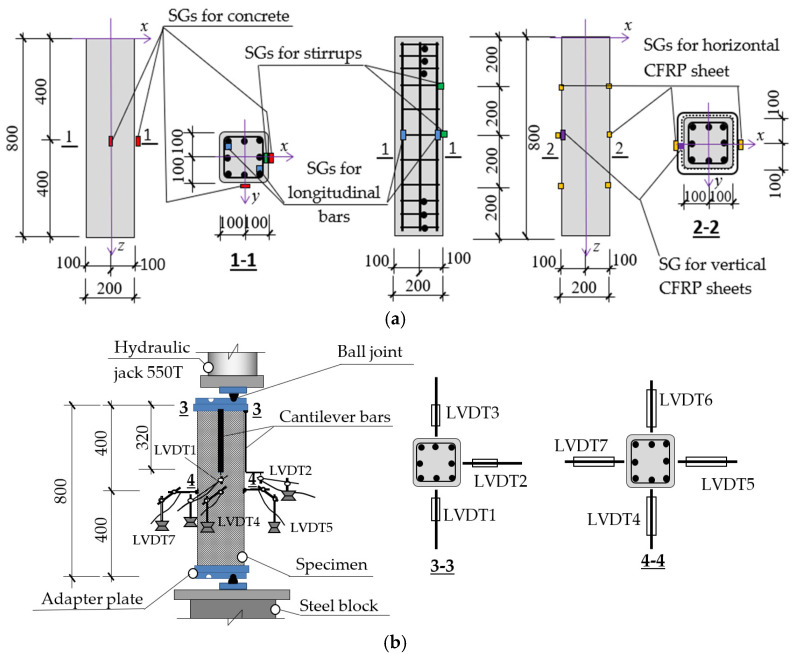
Location of strain gauges and LVDTs (in mm): (**a**) Strain gauges; (**b**) LVDTs.

All the columns were tested with an eccentric loading mechanism (except for column 00-00 under concentric loading), as shown in Figure 5. Figure 6 represents the installed specimen on the loading device. The axial and lateral displacements of the columns were monitored by seven linear variable differential transformers (LVDTs), of which three LVDTs were used for measuring the axial displacement while the remaining four LVDTs were utilized for lateral displacement at the midheight. The compressive strain of concrete was measured by two strain gauges (SGs) attached to the midheight of the columns, as shown in Figure 7. The strain of vertical CFRP sheets was measured by one SG at the extreme tension side, and those of horizontal CFRP sheets were monitored by using six SGs at three sections, including 2 SGs at each section as shown in Figure 7. The strain of longitudinal reinforcements was measured by 2 SGs at the midheight while the strain of stirrups was monitored by 2 SGs attached to midheight and one-fourth of the columns.

## 3. Experimental Results

### 3.1. Failure Mode

**Figure 8:**
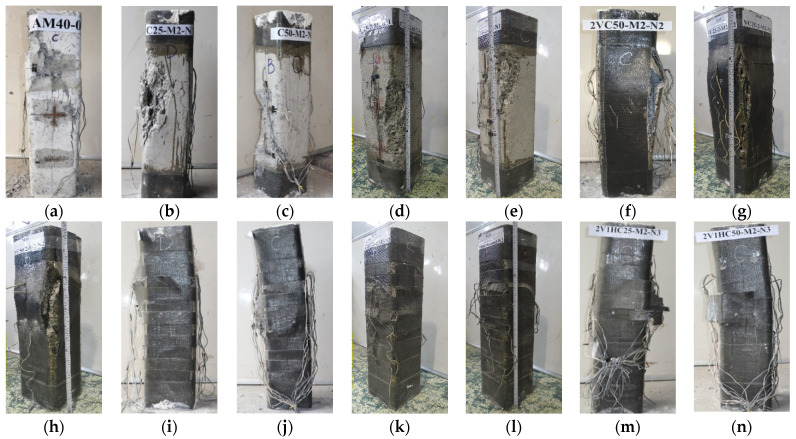
Failure modes of the tested columns; (**a**) 00-00; (**b**) 00-25; (**c**) 00-50; (**d**) 00-2525; (**e**) 00-5050; (**f**) 10C50; (**g**) 10C2525; (**h**) 10C5050; (**i**) 1iC25; (**j**) 1iC50; (**k**) 1iC2525; (**l**) 1iC5050; (**m**) 11C25; (**n**) 11C50.

The failure modes are shown in Figure 8. The unstrengthened columns exhibited three types of failure mode. For specimen 00-00 under concentrated axial load, the first crack appeared at the midheight at 45%*P_u_* (*P_u_* is the ultimate load) and it then developed in the axial direction towards the two ends, leading to final failure due to concrete crushing at the midheight (Figure 8a). As shown in Figure 8b–e, for columns 00-25 and 00-50 under uniaxial loading, the first crack appeared at the flat side of the extreme compression surface around the midheight at 0.40–0.45%*P_u_* and it then developed in the axial direction towards the two ends. Concrete crushing at the midheight caused final failure faster with larger *e/h* ratio. For columns 00-2525 and 00-5050 subject to biaxial loading, the first crack appeared at the extreme compression corner around the midheight at earlier load, about 0.3*P_u_*, and it then developed in the axial direction towards the two ends. Concrete crushing at the corner around midheight caused final failure faster with larger *e/h* ratio. These types of failure mode are appropriate with stress concentration caused by eccentric load (around the midheight at the flat side for uniaxial loading and around the midheight at the corner for biaxial loading).

Figure 8f–h shows the failure modes of laterally unconfined columns which are strengthened only for bending. Cracks appeared at the flat side of the extreme compression surface for uniaxial loading and at the extreme compression corner for biaxial loading. Cracking was heard at 0.40–0.45%*P_u_* and attributed to the local fracture of adhesive associated with the crack formation in concrete. At the ultimate load *P_u_*, CFRP sheets almost completely delaminated at midheight with damaged concrete. The failure happened at the flat side or at the corners. After failure, buckled longitudinal reinforcements were observed as shown in Figure 8f–h. In comparison with unstrengthened columns, longitudinal CFRP sheets delay the crack in the tension surface, while their effects on the compression surface are relatively small, leading to failure around the midheight at the flat side or corners, similar to unstrengthened columns’ failure modes.

The failure modes of partially confined columns with different *e/h* ratio are shown in Figure 8i–l. The first crack appeared at 0.60–0.65*P_u_* around the midheight of the extreme compression surface, and at 0.80*P_u_* local fracture of the adhesive was found with small sound, and the rupture of CFRP sheets at the corner generated the collapse of these columns. The debonded area of the CFRP sheets was larger with higher *e/h* ratio. The stress on the section of partially confined columns was redistributed by transverse and longitudinal CFRP sheets, thus delaying the appearance of the first crack and increasing the ultimate load compare with unstrengthened columns. Before the failure, concrete was crushed but still in the “CFRP-sheet formwork”, so that the columns collapsed immediately at CFRP sheets’ rupture.

The failure modes of full confinement columns with different *e/h* ratio are shown in Figure 8m,n. The first crack appeared at 0.60*P_u_* around the midheight of the extreme compression surface, and at 0.80–0.85%*P_u_* local fracture of adhesive was found with small sound. Once fully confined columns failed, the CFRP sheets partly debonded, aand concrete between longitudinal and transverse reinforcements was partially damaged. However, it was still attached to the buckled reinforcements. This observation was also reported in a previous study on the bond of FRP sheets with various concrete strengths [19]. The debonding occurred inside a thin layer of concrete close to the interface between FRP sheet and concrete.

### 3.2. Load-Displacement Relationship

**Figure 9:**
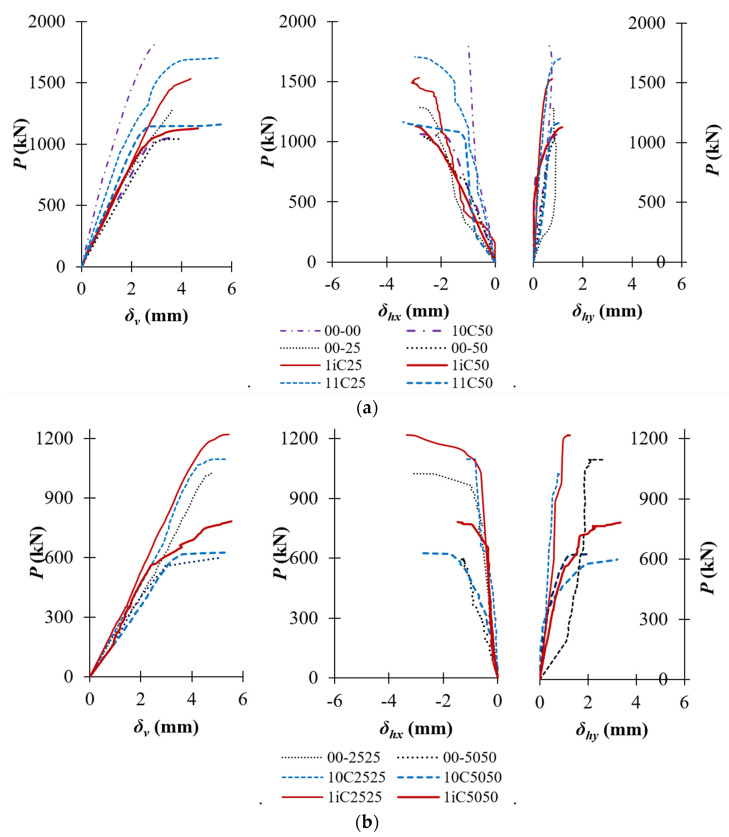
Axial load vs. axial/lateral displacement relationship; (**a**) concentrated and uniaxially loaded columns; (**b**) biaxially loaded columns.

**Figure 10:**
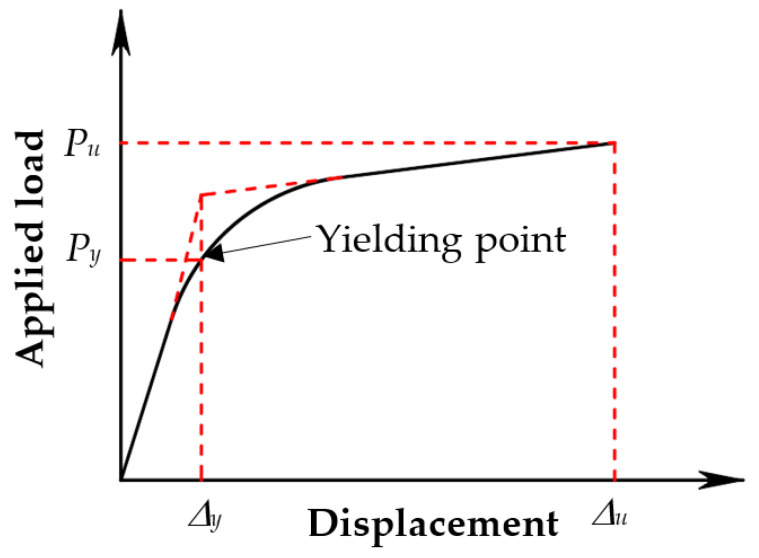
Yielding point definition.

The axial load–displacement curves of the tested columns are represented in Figure 9. A summary of the test results is tabulated in Table 3. *P*_y_ and *P_u_* are yielding and ultimate load, respectively. In Table 3, *P_u_^0^* is the maximum load of the controlled column 00-00. During the early loading stage 0–0.70*P_u_*, the load–displacement curves of the unstrengthened columns were linear, whereas afterward, the columns behaved in a non-linear manner, leading to a significant increase of the displacement. The yielding load, *P*_y_, is defined in Figure 10 as adopted in previous studies [20,21,22]. *P*_y_ was slightly affected by the patterns of CFRP sheets, but it was considerably affected by eccentricity with the value of 64% *P_u_* for concentric load (columns 00-00) and between 84% *P_u_* and 94%*P_u_* for eccentric load as shown in Table 3. For example, the yielding loads of columns 00-25, 00-50, 00-2525, and 00-5050 were respectively 84%, 94%, 94%, and 93% of *P_u_* while the yielding loads of columns 1iC25, 1iC50, 1iC2525, and 1iC5050 were 91%, 88%, 93%, and 84% of *P_u_*, respectively.

An increase of *e/h* ratio significantly reduced column initial axial stiffness (*EA*_0_), defined as the ratio of yielding load and corresponding axial displacement (*δ_y_*). The reduction of the initial axial stiffness of the fully CFRP-confined columns was less than that of the unstrengthened columns (see Table 3). For instance, the decrease of the initial axial stiffness of columns 11C25 and 11C50 was respectively 33% and 40% regarding column 00-00. Meanwhile, the reduction of the initial axial stiffness of unconfined columns 00-25, 00-50, 00-2525, and 00-5050 was much larger and measured at 51%, 55%, 71%, and 75%, respectively (see Table 3). The reduction of axial stiffnesses of the partially CFRP-confined columns and unconfined columns was lower than that of the unstrengthened columns. For example, the axial stiffness reduction of columns 1iC25, 1iC50, 1iC2525, and 1iC5050 were respectively 46%, 50%, 66%, and 71% in comparison with column 00-00. For the same *e/h* ratio, the stiffness of fully and partially CFRP-confined columns was higher than those of unstrengthened columns. The stiffness of columns 1iC50, 1iC2525, and 11C25 was 11%, 17%, and 37% higher than that of columns 00-50, 00-2525, and 00-25, respectively. These observations mean that horizontal CFRP sheets improved column stiffness with the confinement effects.

The reduction of the axial stiffness led to a significant increase in the axial displacement (*δ_v_*) and lateral displacement (*δ_hx_* for x direction and *δ_hy_* for y direction). The higher the *e/h* ratio was, the larger the ultimate displacement of the columns. At the ultimate state, the axial displacement (*δ_vu_*) of unconfined columns 00-25, 00-50, 00-2525, and 00-5050 increased by 23%, 32%, 62%, and 73% in comparison to column 00-00. The corresponding increase of the lateral displacement of unconfined columns 00-25, 00-50, 00-2525, and 00-5050 was up to 238% regarding column 00-00. For the partially CFRP-confined columns, the maximum increment of the axial displacement of 85% was recorded for column 1iC5050 regarding column 00-00. Meanwhile, column 1iC5050 achieved the maximum lateral displacement of 236% in comparison to the reference column 00-00 (Table 3). For the fully CFRP-confined columns, increments of the axial displacement of 82% and 89% were recorded for columns 11C25 and 11C50 regarding column 00-00. As can be seen in Figure 9, at a particular applied load, the displacement increment of the partially and fully CFRP-confined columns was smaller than that of the unconfined columns and the displacement increase also reduced with a higher level of concrete confinement. In addition, the ultimate axial and lateral displacements of the partially and fully CFRP-confined columns were much higher than those of the unconfined columns. For example, the ultimate axial displacements of columns 1iC50 and 11C50 increased by 17% and 43%, and ultimate lateral displacements by 8% and 25% of columns 1iC50 and 11C50 in comparison with column 00-50, respectively. This observation indicated that CFRP sheets were very effective in improving the maximum displacement of the strengthened columns even with partial confinement.

### 3.3. Load-Carrying Capacity and CFRP Strengthening Efficacy

**Figure 11:**
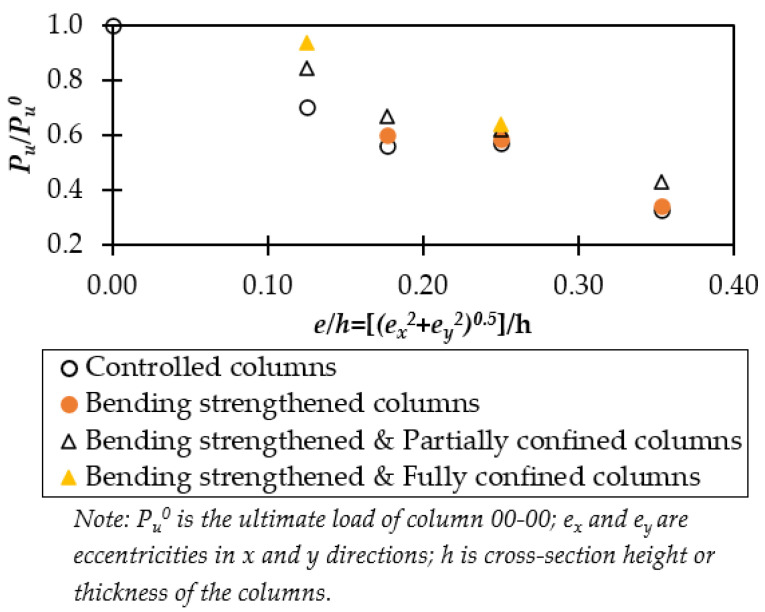
Strength reduction regarding *e/h* ratio.

The increase of *e/h* ratio reduced the axial load-carrying capacity of the unstrengthened columns, e.g., the strength reductions of columns 00-25, 00-50, 00-2525, and 00-5050 were 29%, 43%, 44%, and 67% in comparison to column 00-00, as shown in Table 3. Figure 11 clearly shows the reduction of the load-carrying capacity of the columns when *e/h* ratio increased from 0.12 to 0.35, and the strength reduction was more pronounced for columns with *e/h* ratio of 0.35 as compared to columns with *e/h* ratio of 0.12. For instance, the load-carrying capacity of columns 00-5050 reduced by 67% and columns 00-25 exhibited a reduction of 29% regarding the controlled column 00-00, as shown in Table 3.

The increase of *e/h* ratio also showed a negative effect on the load-carrying capacity of the strengthened columns and the strength reduction is inversely proportional to the level of concrete confinement, e.g., unconfined, partially CFRP-confined, and fully CFRP-confined concrete. Full CFRP-wrap concrete columns increase the stiffness and ultimate displacement of the columns. Thus, the fully CFRP-confined columns experienced less strength loss than the corresponding partially CFRP-confined and unconfined concrete columns. For example, the strength reduction of fully CFRP-confined columns 11C25 and 11C50 was 6% and 36% as compared to the controlled column 00-00 (Table 3) while the strength reduction of the corresponding partially CFRP-confined columns 1iC25 and 1iC50 were 16% and 38%, respectively. At *e/h* = 0.25, the strength reduction of partially CFRP-confined column 1iC50 of 38% was quite closed to that of fully CFRP-confined column 11C50 of 36%. However, when real structures are exposed to a larger *e/h* ratio, there is a need to carry out more research on this topic towards higher *e/h* ratio and larger structures.

**Figure 12:**
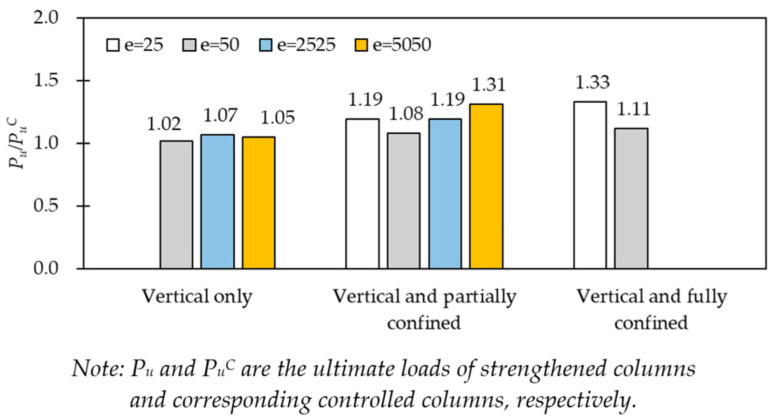
CFRP strengthening efficacy.

The experimental results have also shown that strengthening structures with CFRP sheets is a very effective method in providing lateral confinement to the concrete core. Figure 12 shows that the load-carrying capacity of the strengthened columns was significantly higher than that of the corresponding unstrengthened ones. The load-carrying capacity of the unconfined columns with only flexural strengthening increased by only 2–7%, while the corresponding strength improvement of the partially CFRP-confined columns ranged between 8% and 31%, and that of fully CFRP-confined columns increased 11–33% in comparison with controlled columns, as shown in Table 3. Figure 12 also shows that the difference of strengthening efficacy between partially confined columns and fully confined columns under eccentric load. For instance, the ratios *P_u_*/*P_u_^C^* for partially confined columns 1iC25/00-25 and 1iC50/00-50 were 1.19, and 1.08, respectively, and the ratio *P_u_*/*P_u_^C^* of fully CFRP-confined columns 11C25 and 11C50 were 1.33 and 1.11. Thus, it is worthy to carry out studies to further understand how the partial-CFRP-wrap contributes to the load-carrying capacity of these columns.

### 3.4. Strain of CFRP Sheets and Steel Reinforcements

**Figure 13:**
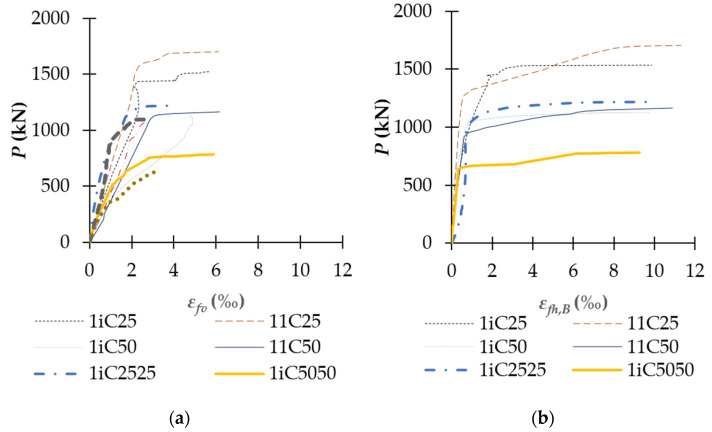
Relationship between the applied load and strain of CFRP sheets: (**a**) Vertical CFRP sheets; and (**b**) Horizontal CFRP sheets.

**Figure 14:**
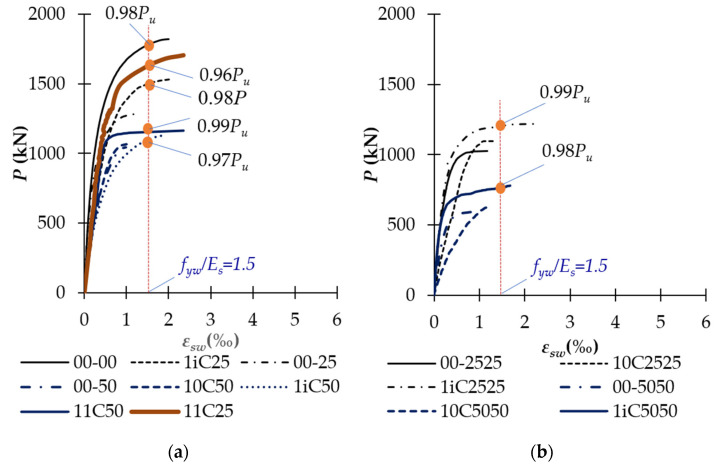
Relationship between the applied load and strain of stirrups: (**a**) Concentrated and uniaxially loaded columns; and (**b**) Biaxially loaded columns.

**Figure 15:**
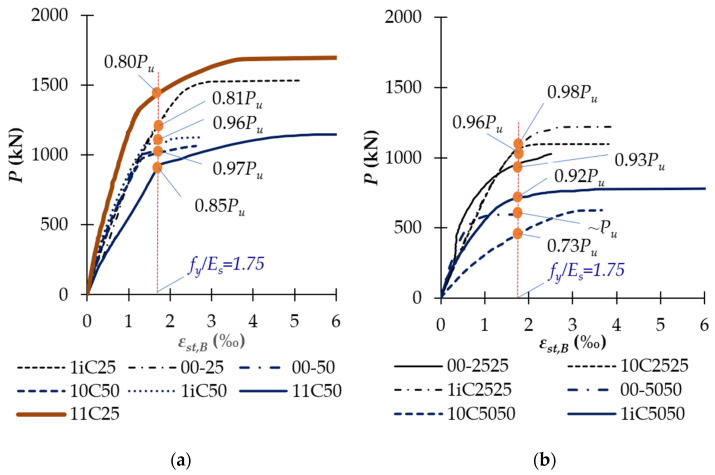
Relationship between the applied load and strain of longitudinal compressive rebars: (**a**) Uniaxially loaded columns; and (**b**) Biaxially loaded columns.

**Figure 16:**
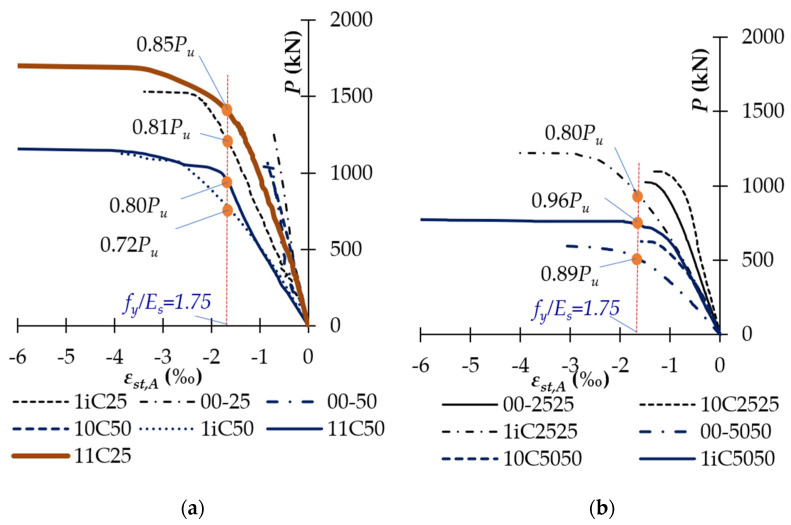
Relationship between the applied load and strain of longitudinal tension rebars: (**a**) Uniaxially loaded columns; and (**b**) Biaxially loaded columns.

Figure 13, Figure 14, Figure 15 and Figure 16 show the relationship between the applied load and strain of CFRP sheets and reinforcements. The strains of rebars in all specimens are tabulated in Table 4. As shown in Figure 13 and Table 4, the ultimate strain of vertical CFRP sheets (*ε_fvu_*) increased with the level of confinement. For example, the ultimate strain of partially CFRP-confined columns 1iC50, 1iC2525, and 1iC5050 increased 81%, 30%, and 81% regarding the corresponding unconfined columns 10C50, 10C2525, and 10C5050, respectively. The increase of vertical-CFRP-sheet ultimate strain of fully CFRP-confined columns were higher, up to 138% for column 11C50 regarding column 10C50. The ultimate strains of transverse CFRP sheets of fully CFRP-confined columns were higher than that of partially CFRP-confined columns. At the extremely compressive side (side B), the increases of *ε_fhu,B_* were about 5% to 18%, and at the extreme tension side (side A), these increases of *ε_fhu,A_* were much higher, up to 21–89%. This phenomenon is due to the better strain distribution of fully CFRP-wrapped columns than partially CFRP-wrapped ones. When *e/h* ratio increases, *ε_fhu,A_* at extremely tension side becomes smaller and approaches zero for both the full and partial wrap. The CFRP strains at ultimate stage were about 0.92% and 0.99% for partially CFRP-wrapped columns and about 1.09–1.13% for fully CFRP-wrapped ones. These ultimate CFRP strains of partially and fully CFRP-wrapped columns correspond to 44–48% and 52–54% of CFRP nominal rupture strain determined from the flat coupon tests, respectively. The range of full wrap columns was inside the range between 51% and 78% of the nominal rupture strain reported by Lam and Teng [23] for fully CFRP-confined square columns, and that of partial wrap columns was slightly lower than the range between 51% and 78%. This observation indicated that partial CFRP-wrap was acceptable in term of developing CFRP tension capacity.

In addition, Figure 14 also shows that stirrups of the unstrengthened columns and unconfined columns did not yield at the ultimate stage. Particularly, stirrups of columns 00-00 under concentric loads yielded at 0.98*P_u_*. This was because the deformation of the unstrengthened and unconfined columns was significantly lower than that of the strengthened columns. Therefore, these columns might fail before yielding stirrups. Meanwhile, stirrups of the partially and fully CFRP-confined columns yielded at the loading level of 0.96–0.99*P_u_* thanks to the confinement effect. These observations have proven the interaction between CFRP and stirrups. The ultimate strain of stirrups of the partially and fully CFRP-confined columns was 1.6–2.0 and 1.8–2.4 times of that of the unstrengthened ones, respectively. These observations demonstrated that the use of partial-CFRP-wrap significantly affected the lateral displacement of the columns.

The longitudinal compressive rebars of the unstrengthened columns yielded at the load level of 0.93–1.00*P_u_* (Figure 15). There, ultimate strain varied between 0.18% and 0.22% and it reduced with high *e/h* ratio. Meanwhile, the partially and fully CFRP-confined columns had longitudinal reinforcements yielding at the load level of 0.81–0.96*P_u_*, and 0.80–0.85*P_u_*, respectively. The ultimate strain of the longitudinal compressive reinforcements of unconfined, partially CFRP-confined, and fully CFRP-confined columns was 1.3–2.1, 1.4–3.5, and 3.6–3.8 times that of corresponding controlled ones, respectively. In addition, Figure 16 also shows that the longitudinal tension rebars of the unstrengthened and unconfined columns did not yield at the ultimate stage. Particularly, longitudinal tension rebars of column 00-5050 yielded at 0.89*P_u_* due high value of *e/h* ratio. This was because the deformation of these columns was significantly lower than that of the strengthened columns. Therefore, these columns might fail before yielding of longitudinal tension reinforcement and it was dependent on *e/h* ratio. As shown in Table 3, the longitudinal tension rebar strain of unconfined columns was even lower, achieving about 48–93% that of the corresponding controlled columns. The reason for this is that vertical CFRP sheets at the tension side work together with tension rebars. Meanwhile, longitudinal-tension-rebars of the partially and fully CFRP-confined columns yielded at the loading level of 0.72–0.96*P_u_* and 0.80–0.85*P_u_*, respectively. The use of CFRP wrap for confinement significantly reduced the axial deformation of the columns and thus delayed the yielding of longitudinal rebars as compared to the unstrengthened and unconfined columns. For example, as shown in Figure 16, longitudinal-tension-rebar of columns 1iC50 and 11C50 yielded at the load level of 0.80*P_u_* and 0.72*P_u_* respectively, while longitudinal-tension-rebar of columns 10C25 and 00-50 did not yield. This is a clue for the interaction between partially and fully CFRP-wrapped and longitudinal-tension-rebar. The ultimate strain of the longitudinal-tension-rebar of partially and fully CFRP-confined columns was 2.1–4.8 and 7.2–9.2 times that of the unstrengthened ones, respectively. These observations demonstrated that both partial and full wrap of CFRP sheets significantly increased the strains of longitudinal rebars.

## 4. Study on Design Guidelines

Two major design guidelines, namely ACI 440.2R-17 [17] of the American Concrete Institute and the Fib Bulletin 14 [18] of the International Federation for Structural Concrete, Switzerland, were selected for reviewing the prediction of CFRP-wrapped RC columns. ACI 440.2R-17 [17] does not provide the information about confinement effect of RC columns partially wrapped with FRP. Meanwhile, Fib introduced a reduction factor for taking into account the effect of partially wrapping columns.

### 4.1. Failure Modes Design Columns According to ACI 440.2R-17

According to ACI 440.2R-17 [17], the ultimate compressive strength of RC columns fully wrapped with FRP under pure axial compression is calculated by Equation (1).

(1) $$\phi P_{n}=0.8\phi \left[0.85f_{cc}^{’}\left(A_{g}-A_{st}\right)+f_{y}A_{st}\right]$$

where *A_g_* (mm^2^) is the cross-section of the columns considering the rounded corner with a radius *R_c_* (mm) = *bh* − (4 − π)*R_c_*^2^, *A_st_* is the cross-sectional area of longitudinal reinforcements, *f’_cc_* is the compressive strength of confined concrete, and *f_y_* is the yield strength of longitudinal reinforcements.

**Figure 17:**
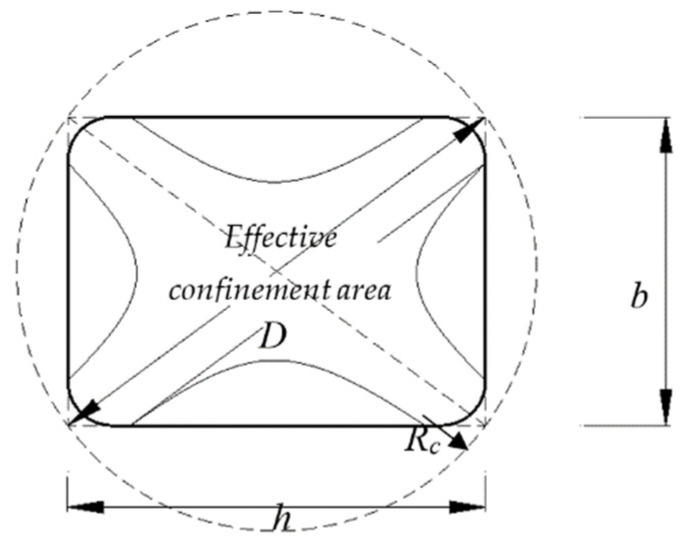
Equivalent circular cross section.

The effective cross-sectional area of concrete *A_e_* (mm^2^) (see Figure 17) is estimated as follows:

(2) $$A_{e}=\frac{1-\left(\frac{\left(\frac{b}{h}\right){\left(h-2R_{c}\right)}^{2}+\left(\frac{h}{b}\right){\left(b-2R_{c}\right)}^{2}}{3A_{g}}\right)-\rho_{g}}{1-\rho_{g}}A_{c}$$

where *A_c_* is the cross-sectional area of concrete and *ρ_g_* is longitudinal steel reinforcement ratio.

The compressive strength of confined concrete, *f’_cc_* (MPa), is calculated as follows:

(3) $$f_{cc}^{’}=f_{co}^{’}+\psi_{f}3.3\kappa_{a}f_{l}$$

where *f’_co_* (MPa) is the compressive strength of unconfined concrete cylinders = 0.8*f_c,cube_*, in which *f_c,cube_* is the compressive strength of concrete cubes, CFRP additional reduction factor *ψ_f_* = 0.95; and $\kappa_{a}$ = (*b*/*h*)^2^ × (*A_e_*/*A_c_*).

The confining pressure, *f_l_* (MPa), is estimated as follows:

(4) $$f_{l}=\frac{2E_{f}\begin{array}{c} \varepsilon_{j}t_{f} \end{array}}{D}$$

where *D* (mm) is the equivalent diameter of rectangular section = (*h*^2^ *+ b*^2^)^1/2^; *E_f_* (MPa) and *ε_j_* are respectively the elastic modulus and nominal rupture of CFRP sheets, and *t_f_* (mm) is the nominal thickness of CFRP sheets.

Models of FRP-confined concrete is defined as the following expressions:

(5) $$f_{c}=\left\{ \begin{array}{l} E_{c}\varepsilon_{c}-\frac{{\left(E_{c}-E_{2}\right)}^{2}}{4f_{c}^{’}}\begin{array}{cc} & 0\leq \varepsilon_{c}\leq \varepsilon_{t}^{’} \end{array}\\ f_{c}^{’}+E_{2}\varepsilon_{c}\begin{array}{cccc} & & & \;\varepsilon_{t}^{’}\leq \varepsilon_{c}\leq \varepsilon_{c.\max} \end{array} \end{array}\right.$$

(6) $$\varepsilon_{c,\max}\leq \varepsilon_{ccu}\leq 0.01$$

(7) $$E_{2}=\frac{f_{cc}^{’}-f_{c}^{’}}{\varepsilon_{ccu}}$$

(8) $$\varepsilon_{t}^{’}=\frac{2f_{c}^{’}}{E_{c}-E_{2}}$$

**Figure 18:**
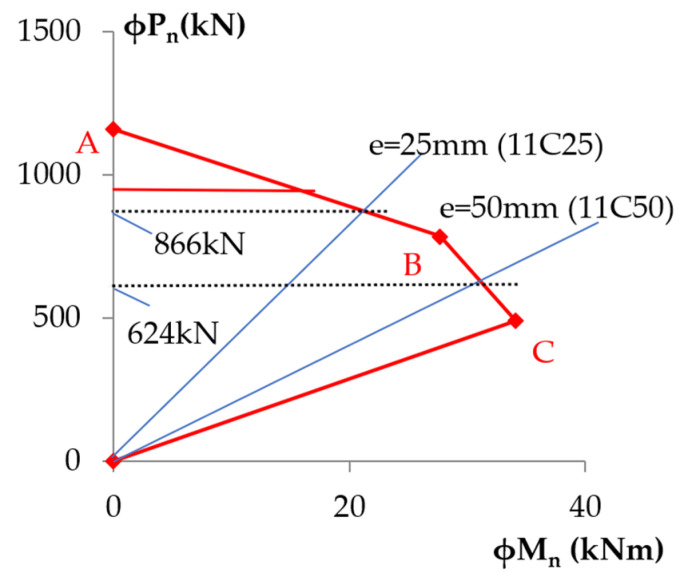
Simplified P-M diagram.

For predicting the effect of FRP confinement on strength enhancement, Equation (1) is applicable when the eccentricity present in the member is less than or equal to 0.1 h. For predicting the effect of FRP confinement on strength enhancement of columns under combined axial compression and bending, P-M diagrams were developed by satisfying strain compatibility and force equilibrium using the model for the stress–strain behavior for FRP-confined concrete presented in Equations (5)–(8). A simplified P-M diagram through three points was used. Equation (1) was applied to locate point A and the methodology provided in Appendix D in ACI 440.2R-17 [17] was used for the computation of a simplified interaction diagram. The ultimate compressive strength of columns11C25 and 11C50 is interpolated as shown in Figure 18.

### 4.2. Design Columns According to Fib Bulletin 14

According to Fib Bulletin 14 [18], the design procedure of FRP-wrap RC columns starts with the calculation of the ultimate confining stress *f_l_* due to the CFRP jacket and can be calculated from Equation (9).

(9) $$f_{l}=\frac{1}{2}\rho_{j}f_{j}=\frac{2t_{j}f_{j}}{d_{j}}$$

where *f_j_* is the ultimate strength of the CFRP jacket and *ρ_j_* is the volumetric ratio of CFRP, being indicated in terms of jacket thickness *t_j_* and equivalent diameter of the column cross section *d_j_*.

The peak load, *f_cc_*, and the peak strain, ε*_cc_*, can be calculated from Equations (10) and (11), respectively.

(10) $$f_{cc}=f_{co}\left(2.254\sqrt{1+7.94\frac{f_{l}}{f_{co}}}-2\frac{f_{l}}{f_{co}}-1.254\right)$$

(11) $$\varepsilon_{cc}=\varepsilon_{co}\left[1+5\left(\frac{f_{cc}}{f_{co}}-1\right)\right]$$

where *f_co_* is the unconfined compressive strength of concrete taken as 49 MPa for the calculation and ε*_co_* is the cracking strain of concrete, taken as 0.002.

Then, the ultimate strength, *f_cu_*, and ultimate strain, ε*_cu_*, can be calculated from Equations (12) and (13).

(12) $$\varepsilon_{cu}=\varepsilon_{cc}{\left(\frac{2\beta \varepsilon_{ju}E_{cc}}{E_{c}-E_{cc}}\right)}^{1-E_{cc}/E_{c}}$$

(13) $$f_{cu}=\frac{E_{c}\varepsilon_{cu}}{1+2\beta \varepsilon_{ju}}$$

where *E_c_* is the elastic modulus of concrete and ε*_ju_* is the ultimate failure strain of the CFRP jacket. *E_cc_* and *β* can be computed from Equations (14) and (15).

(14) $$E_{cc}=\frac{f_{cc}}{\varepsilon_{cc}}$$

(15) $$\beta =\frac{5700}{\sqrt{f_{co}}}-500$$

**Figure 19:**
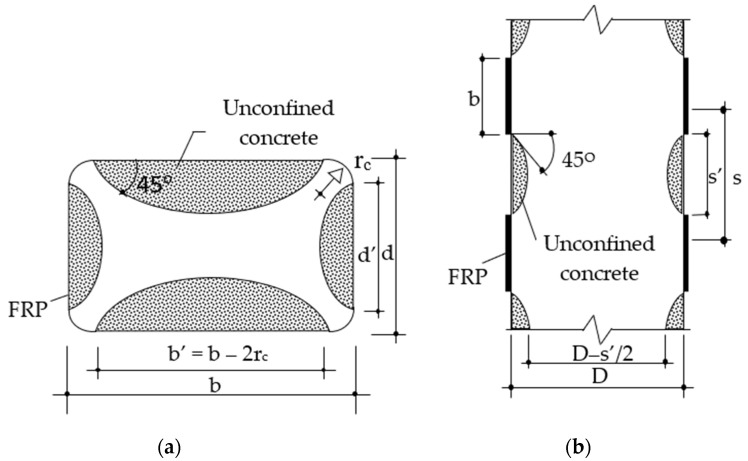
Effective confined area for columns with: (**a**) Rectangular section and (**b**) Partial FRP wrap.

*f_l_* should be modified for non-circular sections and partial confinement by. For rectangular or square columns, modification factor, *k_e_*, is calculated using Equation (16), and for partial confinement modification factor, *k_e_*, is computed as Equation (17) together with the terms elaborated in Figure 19.

(16) $$k_{e}=1-\frac{b{’}^{2}+d{’}^{2}}{3A_{g}\left(1-\rho_{sg}\right)}$$

(17) $$k_{e}=\frac{{\left(1-\frac{s’}{2D}\right)}^{2}}{\left(1-\rho_{sg}\right)}$$

where *ρ_sg_* is the volumetric ratio of longitudinal reinforcement and *A_g_* is the gross cross sectional area.

The axial load carrying capacity of uniaxial-bending columns, *N_Rd_*, can be calculated from Equation (18) derived in accordance with Euro Code 2 [24].

(18) $$N_{Rd}=k_{c}bhf_{cu}$$

where *k_c_* is the maximum value of *N*/(*bhf_ck_*) ratio being derived from the Column Design Chart present in Euro Code 2 depending on the column’s cross section (*b* and *h*), strength (*f_yk_*), total area (*A_s_*), longitudinal steel, concrete ultimate strength taking into account confinement (*f_cu_*), denoted as *f_ck_* in the Chart, d_2_/h = 0.22, and eccentricity (e).

For biaxial-bending columns (1iC2525 and 1iC5050), the strain-compatibility method was used to compute the axial load carrying capacity including the 2nd order effects in each direction per the requirement in Euro Code 2 [24].

### 4.3. Comparison of Experimental Results with Design Guideline Results

The load carrying capacity of tested columns obtained from design calculations and experimental results is shown in Table 5. For full confinement columns, safety factors were between 1.87 and 1.97 when using ACI 440.2R-17 Manual [17], similar to those obtained when using Fib Bulletin 14 Guidelines [18], between 1.97 and 2.09. The differences of safety factors between these two design guides were not much, just 5–6%. However, only Fib Bulletin 14 [18] introduced a modification factor taking into account partially confined columns. The safety factors were from 1.93 to 1.98 for uniaxially loaded columns, and from 1.55 to 1.76 for biaxially loaded ones. The safety factors of biaxially loaded columns were 9–21% lower than that of uniaxially loaded columns. Therefore, more studies on this topic are deemed necessary to provide more useful data and analysis so that the design of partially FRP-confined columns can be applied with high confidence.

## 5. Conclusions

This study investigated the performance of rectangular RC columns with different patterns of CFRP-wrap subject to uniaxial and biaxial load in order to evaluate the CFRP strengthening efficiency of partially and fully confined RC columns under uniaxial and biaxial load, and to study the existing guidelines on CFRP confinement design to establish the safety factor of the predicted load-carrying capacity against the experimental results. The main findings can be summarized as follows:

(1) Both partial and full CFRP wrapping demonstrated effectiveness in improving axial stiffness, load-carrying capacity, and optimization of reinforcement strains:
  - Initial axial stiffness of strengthened columns increased up to 17% and 37% for partially and fully CFRP-confined columns in comparison with those of corresponding controlled columns, respectively. As a result, the ultimate axial and lateral displacement of partially CFRP-confined columns increased up to 19% and 53%, and those of fully CFRP-confined columns increased up to 48% and 62%, respectively;
  - Load-carrying capacity of strengthened columns increased up to 31% and 33% for partially and fully CFRP-confined columns in comparing with those of corresponding controlled columns, respectively.
  - The ultimate strain of horizontal CFRP sheets varied between 0.92% and 1.13%, corresponding to 44–54% of their rupture strain from coupon tests. The difference between the CFRP strain of partial wrap columns and that of full wrap ones was small. The use of horizontal CFRP sheets delayed the yielding of compressive reinforcements and thus increased the stiffness of confined columns. Horizontal CFRP sheets also improved the ultimate strain of stirrups and longitudinal compressive rebars from 1.6 to 2.4 times and 1.4 to 3.8 times, respectively.
  - The ultimate strain of vertical CFRP sheets increased with the increase of e/h ratio as well as confined level of concrete, and it varied 0.26% and 0.62%. The use of CFRP sheets delayed the yielding of tension reinforcements, and this effect is bigger with a higher level of confinement.
(2) From the comparison with test results and predicted strengths by code equations, for full confinement columns, safety factors were between 1.87 and 1.97 when using ACI 440.2R-17 Manual [17], similar to those obtained when using Fib Bulletin 14 Guidelines [18], between 1.97 and 2.09. The differences in safety factors between these two design guides were not much, just 5–6%. However, for partially confined columns, the strength prediction is possible only in Fib Bulletin 14 [18] and the safety factors evaluated by using it were from 1.93 to 1.98 for uniaxially loaded columns and from 1.55 to 1.76 for biaxially loaded ones. The safety factors of biaxially loaded columns were 9–21% lower than that of uniaxially loaded columns. Therefore, more studies on this topic are deemed necessary to provide more useful data and analysis so that the design of partially FRP-confined columns can be applied with high confidence.

## Figures and Tables

**Table 1:** Mix design and mechanical properties of concrete.

Grade	Cement kg/m^3^	River Sand kg/m^3^	Coarse Aggregates kg/m^3^	Fine Aggregates kg/m^3^	Water L/m^3^	Superplasticizer L/m^3^	*f_c,cube_* Mpa	*f_sp,cube_* Mpa	Slump mm
M40	414	802	788	263	185	4.0	49	4.5	125

*f_c,cube_* is compressive strength of unconfined concrete cubes, and *f_sp,cube_* is splitting tensile strength of concrete from concrete cubes.

**Table 2:** Mechanical properties of adhesive, CFRP sheets, and steel reinforcement.

	Impregnation *		CFRP				Longitudinal Rebars			Stirrups		
	*f_adhesive,u_* Mpa	*E_adhesive_* Gpa	*f_fu_* Mpa	*ε_fu_* %	*t_f_* * mm	*E_f_* Gpa	*f_u_* Mpa	*f_y_* Mpa	*E_s_* Gpa	*f_uw_* Mpa	*f_yw_* Mpa	*E_s_* Gpa
Mean	60	3-3.5	3579	2.08	0.166	201	621	350	200	470	303	200
COV (%)	-	-	16	10	-	8	3	1	2	2	1	2

* provided by the manufacturer.

**Table 3:** Test result.

Columns	*P_u_*	*P_u_/P_u_* ^0^	*δ_vu_*	*δ_hxu_*	*δ_hyu_*	*EA*_0_	*P_y_*	*E_p_*
	(kN)	(%)	(mm)	(mm)	(mm)	(kN/mm)	(kN)	(kNmm)
00-00	1819	100	3.0	−1.0	0.6	767	1156	3234
00-25	1284	71	3.7	−2.8	0.7	373	1077	2623
00-50	1044	57	4.0	−2.7	0.9	347	985	2593
00-2525	1025	56	4.9	−3.1	0.7	224	963	2539
00-5050	597	33	5.2	−1.2	3.2	190	556	2125
10C50	1064	58	3.8	−2.8	0.9	375	915	2539
10C2525	1096	60	5.3	−1.2	2.7	255	1022	3340
10C5050	625	34	5.4	−2.9	2.0	182	583	2281
1iC25	1533	84	4.4	−2.8	0.8	413	1402	3876
1iC50	1128	62	4.7	−2.9	1.2	385	990	3655
1iC2525	1219	67	5.5	−3.3	1.1	261	1137	3819
1iC5050	782	43	5.6	−1.5	3.4	220	660	2798
11C25	1704	94	5.5	−3.0	1.1	512	1450	6576
11C50	1164	64	5.7	−3.4	1.0	462	1079	5120

**Table 4:** Strains at ultimate load.

Columns	*P_u_*	*ε_stu,A_*	*ε_stu,B_*	*ε_swu_*	*ε_cu_*	*ε_fhu,A_*	*ε_fhu,B_*	*ε_fvu_*
	(kN)	(‰)	(‰)	(‰)	(‰)	(‰)	(‰)	(‰)
00-00	1819	2.2	1.7	2.0	3.2			
00-25	1284	−0.7	1.9	1.3	3.4			
00-50	1044	−1.0	1.9	1.0	3.6			
00-2525	1025	−1.5	2.5	1.2	3.1			
00-5050	597	−3.3	1.8	1.0	3.2			
10C50	1064	−0.8	2.6	1.0	3.5			2.6
10C2525	1096	−1.4	3.8	1.3	3.5			2.8
10C5050	625	−1.6	3.7	1.2	3.3			3.3
1iC25	1533	−3.4	5.2	2.0	4.2	2.6	9.9	5.7
1iC50	1128	−3.9	2.8	1.9	4.1	4.0	9.7	4.7
1iC2525	1219	−4.1	4.0	2.3	4.3	4.6	9.9	3.7
1iC5050	782	−6.9	6.2	1.7	3.6	4.0	9.2	5.9
11C25	1704	−6.6	7.5	2.4	4.5	4.9	11.3	6.1
11C50	1164	−7.1	7.0	2.4	4.2	5.8	10.9	6.2

**Table 5:** Comparison of experimental results vs. design calculations.

Specimens	*P_u_* (kN)			Safety Factor	
	Exp	Fib	ACI	*P_u,Exp_*/*P_u,Fib_*	*P_u,Ex_*_p_/*P_u,ACI_*
1iC25	1532.9	792.9	-	1.93	-
1iC50	1128.2	569.0	-	1.98	-
1iC2525	1219.0	693.2	-	1.76	-
1iC5050	781.9	504.8	-	1.55	-
11C25	1703.8	817.0	866.4	2.09	1.97
11C50	1163.6	592.0	623.6	1.97	1.87

## Data Availability

All data, models, and code generated or used during the study appear in the published article.
